# Reply to “Key Points to Consider When Evaluating Andexxa for Formulary Addition”

**DOI:** 10.1007/s12028-020-01008-z

**Published:** 2020-06-22

**Authors:** Charles E. Mahan

**Affiliations:** University of New Mexico College of Pharmacy, Presbyterian Healthcare Services, 1100 Central Ave SE, Albuquerque, NM 87106 USA

Dear Editor,

I read with interest the article evaluating ANDEXXA (coagulation factor Xa [recombinant], inactivated-zhzo; andexanet alfa [US-adopted name]) for formulary addition by Peled et al. [1]. However, many statements in the publication require further clarification, which I address below.

## Standard of Care

Peled et al. state that no studies compare andexanet alfa to the standard of care, four-factor prothrombin complex concentrates (4F-PCCs). However, 4F-PCCs are not the standard of care for direct oral anticoagulant (DOAC)-associated bleeding, nor are they Food and Drug Administration (FDA)-approved for this indication. 4F-PCCs were developed and approved specifically to replace depleted coagulation factors in patients receiving warfarin. Andexanet alfa is FDA approved for the reversal of factor Xa (FXa) inhibitor-associated bleeding. Moreover, the FDA recognized that there was an “unmet medical need” not adequately addressed by available therapy and therefore granted accelerated approval [2].

Although 4F-PCCs may constitute usual care in some hospitals, the data available to support its use are of a low quality of evidence, as the article authors noted. Given this low-quality evidence, several guidelines suggest the use of 4F-PCCs to reverse apixaban- or rivaroxaban-associated major bleeding, only if andexanet alfa is not available [3–8]. No prospective, randomized controlled studies have been conducted in bleeding patients to evaluate the efficacy of 4F-PCCs for reversal of anticoagulation with FXa inhibitors.

## Pharmacology

Andexanet alfa is a specific, rationally designed reversal agent that demonstrated a rapid and profound reduction in unbound drug concentration, anti-fXa activity, and restoration of thrombin generation [9]. While the elimination half-life of andexanet alfa may be shorter than that of FXa inhibitors, other key points should be considered. First, experts hypothesize that andexanet alfa’s ability to bind the inhibitor and reverse its anticoagulant activity allows sufficient time to establish a hemostatic plug. This resulted in a 12-h hemostatic efficacy rate of 82% in the ANNEXA-4 study [10]. Peled et al. state that “anti-fXa activity starts to resume to baseline after the 2-h infusion and goes back to the baseline by 4 h after drug initiation.” In fact, anti-fXa levels did not return to baseline in the ANNEXA-A/R studies, but were similar to placebo 4 h after bolus initiation in patients who received the infusion [9]. In ANNEXA-A/R and ANNEXA-4, anti-fXa levels were significantly lower than baseline throughout the 2-h infusion, and in ANNEXA-4, median percent reductions in anti-fXa activity for the rivaroxaban and apixaban groups were 90% and 92% at end of infusion and 42% and 32% at 4 h post-infusion [10].

Second, in response to the comments in Peled et al. on tissue factor pathway inhibitor (TFPI), 4F-PCCs appear to have significantly longer effects on endogenous thrombin potential compared with andexanet alfa [9, 11]. Peak thrombin generation was also substantially higher with 4F-PCC than with andexanet alfa [9, 11]. Andexanet alfa–TFPI interaction has only a small effect on thrombin generation, which affects only the extrinsic (not intrinsic) pathway [12].

Third, Peled et al. suggest that andexanet alfa may increase the risk of thrombosis. In the ANNEXA-A/R studies in healthy volunteers, no thrombotic events occurred [9]. However, bleeding patients taking FXa inhibitors are at higher risk for both recurrent bleeding and thrombotic events, which may reflect the patient population rather than the treatment. In the ANNEXA-4 study in bleeding patients, there were no thrombotic events after the restart of oral anticoagulation [10], suggesting that these events are more likely associated with returning high-risk patients to their baseline thrombotic risk while not taking an anticoagulant. Similarly, thromboembolic events occurred in FXa inhibitor trials when patients transitioned off rivaroxaban and apixaban [13, 14]. It is also possible that thrombotic events were more likely to be reported in ANNEXA-4, which followed patients for 30 days, versus PCC studies with a shorter follow-up period (e.g., chart review studies that track events through hospital discharge).

Lastly, in bleeding patients taking DOACs, PCC dosing ranges from ~ 10 to 160 units/kg with little standardization [6, 15]. A recent study by Green et al. [16] reported significantly increased mortality in patients with DOAC-associated bleeding treated with PCCs at doses > 25 units/kg—suggesting the possibility that PCCs may be more harmful than beneficial in this population without clear dosing trials. Indeed, emerging comparative data suggest that 30-day mortality rates were 19.5% lower with andexanet alfa versus PCC across all bleed types (14.6% vs 34.1%; *P *< 0.001; RRR of 57.2%) and 33.6% lower (15.3% vs 48.9%; *P *< 0.001; relative risk reduction [RRR] of 68.7%) for the most feared bleed type, intracranial hemorrhage [17].

Peled et al. note the FDA clinical reviewers’ concerns regarding the safety and efficacy of andexanet alfa, but did not include the prediction of benefit by the Division Director, who oversaw the review process. The Director believed that andexanet alfa’s effect on the surrogate outcome was reasonably likely to predict a clinical benefit due to its > 90% decrease in anti-fXa activity, and the strong biological plausibility that this decrease could lead to hemostasis and improve morbidity and mortality in bleeding patients receiving apixaban and rivaroxaban who require reversal [2].

Peled et al. also state that the ANNEXA-I trial (NCT03661528) was designed to compare andexanet alfa versus standard of care, when in fact ANNEXA-I compares andexanet alfa versus “usual care,” which includes any investigator-prescribed commercially available product. 4F-PCCs will likely be one of many therapies in the usual care arm. Data from a recent real-world study of electronic medical records from 3030 patients hospitalized for FXa-associated bleeding showed that 24% of cases were managed with 4F-PCC [18].

## PCC Study Limitations

A recent systematic review on PCCs by the American Society of Hematology stated that it was currently “impossible to know whether 4F-PCC is more effective than supportive care alone” and that controlled studies are needed to make this determination [19]. The review also noted that there was a “very serious risk of bias,” specifically confounding and selection bias; outcome assessors in all studies were not blinded; only 2 of 10 studies included consecutive patients; evidence imprecision was rated as very serious given the small sample sizes and very few events; and, importantly, certainty of evidence was very low. Of the 10 studies included in the systematic review, only 150 to 249 patients were included in the pooled analyses, far fewer than the ANNEXA-4 study of 352 patients [10]. Further, many patients in the 4F-PCC studies may not have had therapeutic FXa activity levels since this was not measured in the majority. For example, 50% of patients in the Schulman et al. study had no clinical evidence of therapeutic anticoagulation [20], which suggests bleeding control would have been the same without PCC intervention. Of note, this study excluded patients with do-not-resuscitate orders, and patients who were re-dosed or required surgery were not rated as having poor/none hemostatic efficacy.

## Off‐label Use of PCCs

The authors discuss the off-label use of amiodarone, which has a very plausible mechanism for the treatment of atrial fibrillation; however, this is not analogous to the use of 4F-PCCs to treat major bleeding in patients taking FXa inhibitors. 4F-PCCs are not a single drug but a mix of multiple factors, most with no plausible mechanism or effect as a nontargeted factor replacement strategy for FXa inhibitor-associated bleeding, increasing the likelihood of off-target effects. Prior to the approval of andexanet alfa, there were no other treatment options available. Due diligence suggests that clinicians select a drug with a clearly demonstrated mechanism of action and FDA approval.

## Society Guidelines, Conflict of Interest, and Bias

Peled et al. discuss conflicting society guidelines for the management of DOAC-related bleeding. While guidelines take years to synthesize and publish, they often become quickly obsolete given the rapid introduction of new studies and data. Many guidelines were written before the availability of andexanet alfa and final ANNEXA-4 study results, and are currently under revision. More recent guidelines are placing andexanet alfa as a first-line agent in front of PCC [3–8].

The authors also note that the conflicts of interest declared by guideline writers must be considered. I agree that disclosure of potential conflicts of interest is important to ensure that decision-makers are independent with minimal conflict as related to industry and sponsors. However, data show that 84% of physicians have had some relationship with industry [21]. At the end of the day, industry and national organizations typically rely on experts for advice and research to optimize the development of critical medications and develop guidelines. Excluding those most knowledgeable about the medication and therapeutic area would minimize the impact of the guidelines.

Importantly, an individual may be free from conflict, but not free from bias. In fact, conflicts of interest are frequently declared, but biases are not. Studies note cognitive biases in healthcare providers, including physicians and pharmacy and medical directors that serve on pharmacy and therapeutics committees [22, 23]. Cognitive biases are tendencies to process information in patterns that may interfere with rational healthcare decision-making, leading to misinterpretation of clinical information and suboptimal formulary choices. Several types of bias may affect healthcare decisions, including overconfidence, tolerance to risk/ambiguity, confirmation bias, anchoring bias, relative versus absolute framing effect, risk aversion, zero-risk bias, and delay discounting [22, 23].

## Financial Impact

Peled et al. compared the costs of PCCs and andexanet alfa, yet further clarification is needed. First, the current wholesale acquisition cost (WAC) per unit of 4F-PCCs is $2.50 (Micromedex, 2020), 54% higher than the $1.62 noted by Peled et al. Second, the average US male and female weigh 90 kg and 77 kg, respectively [24]. Utilizing a weight of 85 kg, the cost of 4F-PCCs with WAC and doses of 25 to 100 units/kg would range from $5313 to $21,250. The WAC for andexanet alfa is $27,500 for the low dose and $49,500 for the high dose (Micromedex, 2020). Of note, the lower dose was used in ~ 85% of patients in ANNEXA-4 [10]. Third, Peled et al. did not discuss the New Technology Add-on Payment (NTAP); andexanet alfa-specific C-code and J-codes, which allow for pass-through pricing in an outpatient environment [25]; 340b drug pricing; or the availability of andexanet alfa on consignment. As a supplement to the Medicare Severity-Diagnosis Related Group (MS-DRG) payment, NTAP may facilitate an additional payment equal to the lesser of (1) 65% (up to $18,281) of the cost of andexanet alfa, or (2) 65% (up to $18,281) of the amount by which the costs of the case exceed the standard MS-DRG payment. A recent budget impact model showed cost reductions with andexanet alfa versus 4F-PCCs when including both NTAP and the in-patient consequences associated with hematoma expansion (e.g., intubation) [26]. Thus, andexanet alfa pricing and financial impact can be highly variable, and the cost of 4F-PCCs may be similar to that of andexanet alfa, depending on 4F-PCC dosing.

## Summary

4F-PCCs have provided a temporary, nonspecific treatment for FXa inhibitor-associated bleeding prior to specific antidote availability. 4F-PCCs have not been accepted as the standard of care given the lack of consistent evidence, lack of clinical development, lack of effect on anti-FXa levels, and lack of standardized dosing. Further, 4F-PCCs may provide no benefit beyond merely stopping the FXa inhibitor and providing supportive care. In addition, mortality appears to increase with use of PCCs at higher doses.

Andexanet alfa has consistent data from several clinical trials and a committed clinical trial program with new studies addressing the comparator of usual care, as well as a surgical trial (ANNEXA-S; NCT04233073). Andexanet alfa has the mechanism, FDA approval, and strongest data available, with ongoing clinical development, to warrant consideration for addition to a hospital formulary.
